# An immunoscore nomogram for predicting survival in patients with oesophageal cancer

**DOI:** 10.18632/aging.202686

**Published:** 2021-03-10

**Authors:** Gaoming Li, Qiuyue Song, Angsong Cai, Zeliang Wei, Rui Zhang, Dali Yi, Jia Chen, Fang Li, Yanqi Zhang, Ling Liu, Yazhou Wu, Dong Yi

**Affiliations:** 1 Department of Health Statistics, Army Medical University, Chongqing, China; 2 Department of Journal Editorial, Army Medical University, Chongqing, China

**Keywords:** oesophageal cancer, CIBERSORT, immune cell, prognosis, nomogram

## Abstract

This study aimed to construct and validate an immunoscore nomogram that may be used to predict the prognosis of oesophageal cancer. With the gene expression data of oesophageal cancer in a public database, we used CIBERSORT to estimate the fractions of 22 infiltrating immune cell types. We then built an immunoscore signature based on 12 types of infiltrating immune cells using the least absolute shrinkage and selection operator (LASSO) model. This immunoscore was used as an independent predictor in the prognostic model (training cohort: [hazard ratio (HR), 4.78; 95% confidence interval (CI), 2.64-8.67; P < 0.001], validation cohort: [HR, 2.15; 95% CI, 1.04-4.45; P = 0.040]). Subgroup analysis by clinical features showed that overall survival was significantly different between the high-immunoscore group and the low-immunoscore group. The predictors that constituted the individualized prediction nomogram were immunoscore, age, and tumour stage. The nomogram had good discrimination and calibration. Decision curve analysis showed that the immunoscore nomogram was clinically useful. Therefore, the novel immunoscore signature based on infiltrating immune cells can be used as a reliable predictor of the prognosis of oesophageal cancer, and the immunoscore nomogram is a convenient tool for predicting the survival of individual patients.

## INTRODUCTION

As the sixth most common cancer, oesophageal cancer is one of the most aggressive diseases in the world. Oesophageal squamous-cell carcinoma and oesophageal adenocarcinoma are two common subtypes of oesophageal cancer. While the prognosis of oesophageal cancer has been improving in the past few decades, the 5-year survival rate is only 20%, which is still worse than that of many other cancers [1, 2]. Clinically, tumour stage is the primary predictor of oesophageal cancer survival, but patients in the same stage and who are receiving similar treatments may have vastly different outcomes. The immune status in the tumour microenvironment is related to tumour development, patient survival, and treatment response, indicating that tumour-infiltrating immune cells may be a new potential predictor of the prognosis of oesophageal cancer [3, 4].

Extensive research on the effect of tumour-infiltrating immune cells on the prognosis of oesophageal cancer [5, 6] has shown that immune cell infiltration, especially the immunoscore signature based on multiple markers, may be another predictor in addition to tumour stage [7, 8]. However, the efficacy of the immunoscore signature in predicting the prognosis of oesophageal cancer relative to that of traditional predictors is unknown. Traditional methods of evaluating tumour immune infiltration include immunohistochemistry and flow cytometry. With the current technology, the ability of immunohistochemistry to simultaneously measure multiple immune markers is limited, complicating comprehensive evaluations of the immune effects of different cell types or closely related cell populations. For flow cytometry, tissue must be broken down, which may cause cell loss or damage and affect the results. Another method is to obtain high-dimensional data of gene expression profile from the cell mixture, which are then used to deduce the fraction of immune cells [9]. This approach is independent of surface markers and cell division-related human factors.

In this study, we used “cell-type identification by estimating relative subsets of known RNA transcripts (CIBERSORT)” to quantify the fraction of immune cells [10]. This is a deconvolution approach that uses a priori knowledge of expression data to deduce the fractions of 22 immune cell types. CIBERSORT has been used to evaluate the prognosis of various cancers and is considered the most accurate method of cell-type identification [11, 12]. We used CIBERSORT to calculate the fraction of immune cells in tumour and normal tissues based on the gene expression data of oesophageal cancer from a public database. We identified an immunoscore signature based on 12 types of infiltrating immune cells. This signature can be used as a biomarker to predict the overall survival of patients with oesophageal cancer. We also validated its prognostic value in an independent cohort.

## RESULTS

### Clinical characteristics of study cohorts

**Figure 1:**
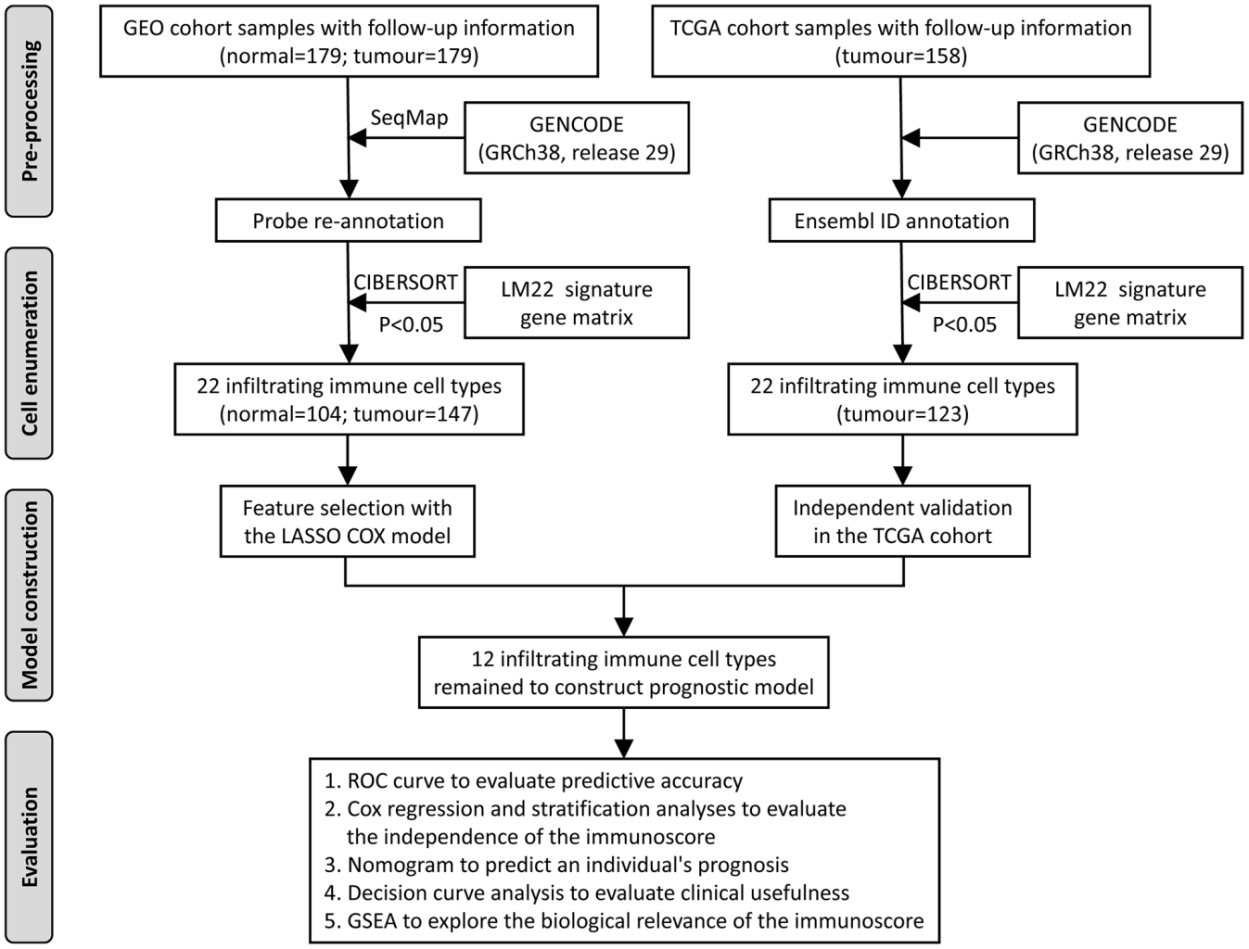
**Flowchart of the study design.** GEO, Gene Expression Omnibus; TCGA, The Cancer Genome Atlas; ROC, receiver operating characteristic; GSEA, gene set enrichment analysis.

An overview of this study is delineated in Figure 1. The clinical information of the two oesophageal cancer cohorts was downloaded from the public Gene Expression Omnibus (GEO) and The Cancer Genome Atlas (TCGA) databases. Based on the inclusion criterion (CIBERSORT P < 0.05), a total of 374 samples were included in this study, including 104 normal samples from the GEO database and 270 cancer samples from GEO (147) and TCGA (123). The cancer samples from the GEO database were the training cohort to identify prognostic markers and construct the model, which was then validated with the TCGA data. In the training cohort, the median age at diagnosis was 60.0 (54.0-66.0) years, and 79.6% of the patients were men. In the validation cohort, the median age at diagnosis was 60.0 (54.0-73.0) years, and 82.9% of the patients were men. The detailed clinical information of these two groups is shown in Table 1.

**Table 1:** Clinical and pathological characteristics of the training and validation cohorts of oesophageal cancer patients.

**Characteristics**	**Training cohort** **(n = 147)**	**Validation cohort** **(n = 123)**	**χ^2^**	**P value**
Age			0.657	0.418
<60	73 (49.7)	55 (44.7)		
≥60	74 (50.3)	68 (55.3)		
Sex			0.486	0.486
Female	30 (20.4)	21 (17.1)		
Male	117 (79.6)	102 (82.9)		
Alcohol			7.603	0.006*
Yes	83 (56.5)	88 (71.6)		
No	64 (43.5)	33 (26.8)		
Missing		2 (1.6)		
Tumour grade			1.683	0.431
G1	24 (16.3)	12 (9.8)		
G2	83 (56.5)	51 (41.5)		
G3	40 (27.2)	33 (26.8)		
Missing	0	27 (21.9)		
Tumour stage			13.633	< 0.001*
T1 + T2	32 (21.8)	48 (39.0)		
T3 + T4	115 (78.2)	63 (51.2)		
Missing	0	12 (9.8)		
Lymph node stage			4.393	0.036*
N0 + N1	120 (81.6)	100 (81.3)		
N2 + N3	27 (18.4)	10 (8.1)		
Missing	0	13 (10.6)		
Pathologic tumour stage			3.756	0.053
I + II	73 (49.7)	68 (55.3)		
III + IV	74 (50.3)	42 (34.1)		
Missing	0	13 (10.6)		

* P < 0.05.

### Differential immune cell fraction

**Figure 2:**
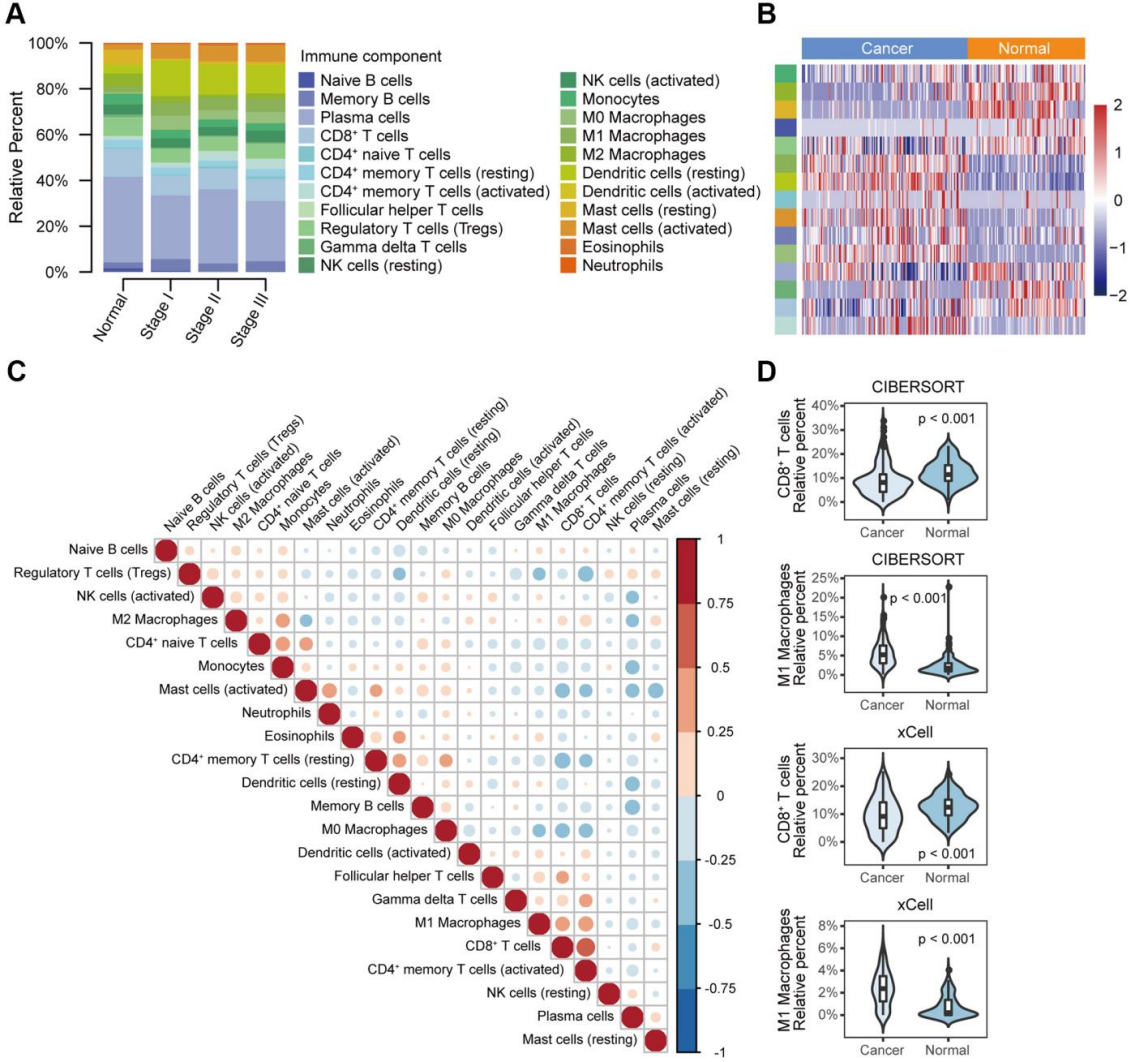
**Different immune cell profiles between normal tissue and cancer tissue.** (**A**) Relative immune cell fractions in the normal and cancer groups estimated with CIBERSORT based on gene expression profile data (GSE53625). (**B**) Heat map of differential immune cell fractions between the normal and cancer groups (FDR < 0.05). P values were calculated with the Mann–Whitney U test and adjusted for multiple testing (FDR). The left side bar shows the type of immune cells in A. (**C**) Correlation matrix of 22 immune cell types in the cancer group. Correlation coefficients were used to sort the cells by hierarchical clustering. (**D**) Violin plots of the abundance of CD8^+^ T cells and M1 macrophages calculated with CIBERSORT and xCell. The box plots in the violin indicate the median and interquartile range of the data distribution.

We first compared the relative abundance of 22 immune cell types between normal and oesophageal cancer tissues from the GEO database. In general, the top five immune cells in cancer and normal tissues were plasma cells, CD8^+^ T cells, dendritic cells (resting), regulatory T cells (Tregs), and mast cells (activated); the total fraction averaged at 64.8% in cancer tissues and 64.0% in normal tissues (Figure 2A). Moreover, dendritic cells (resting), M0 and M1 macrophages, mast cells (activated), memory B cells, CD4^+^ naive T cells, and CD4^+^ memory T cells (activated) were significantly enriched in cancer tissues relative to normal tissues, while mast cells (resting), M2 macrophages, naive B cells, CD8^+^ T cells, gamma and delta T cells, plasma cells, Tregs, and monocytes were significantly reduced in cancer tissues (Figure 2A, 2B, 2D). Immune cell fractions calculated with xCell showed similar results. The analysis validated the reliability of the CIBERSORT results and showed that the immune cell profile could be used as a potential prognostic marker, as it effectively distinguished normal tissue and cancer tissue (Figure 2D). Correlation analysis showed weak to moderate correlations between different immune cell types in the cancer group. CD8^+^ T cells were positively correlated with CD4^+^ memory T cells (activated) (r = 0.65, P < 0.001) and were negatively correlated with CD4^+^ memory T cells (resting) (r = -0.46, P < 0.001) (Figure 2C).

### Feature selection and immunoscore calculation

**Figure 3:**
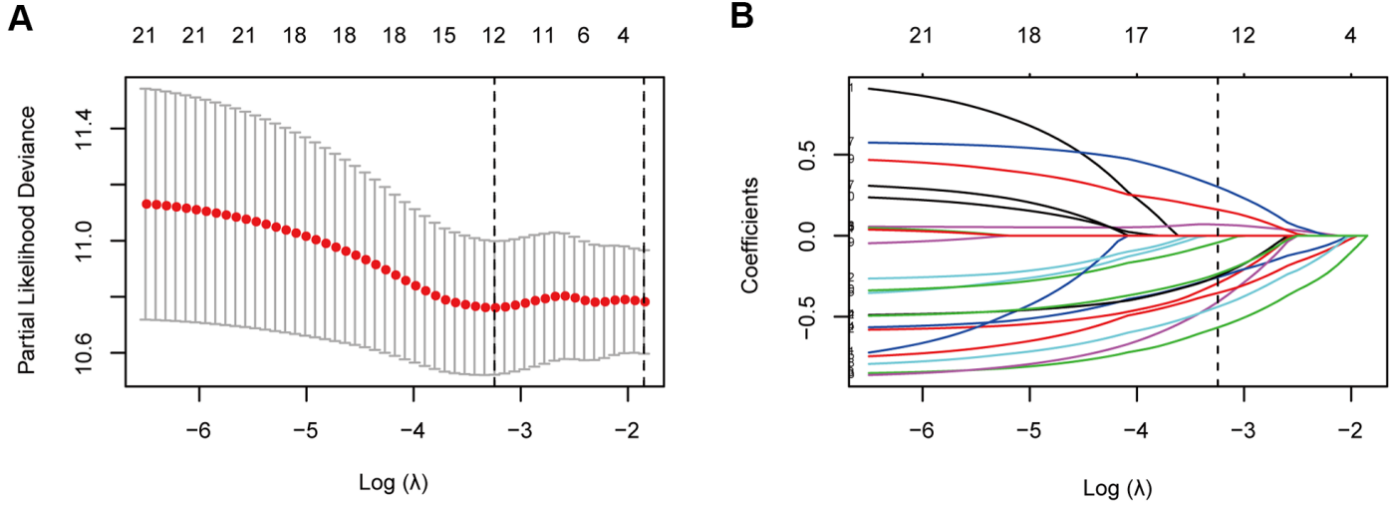
**Immune feature selection based on the LASSO Cox regression model.** (**A**) Optimal feature subsets based on the minimum criteria with 10-fold cross-validation of the LASSO regression. The vertical dashed lines represent the optimal values using the minimum criteria and the 1-standard error (SE) of the minimum criteria. The λ selected with 10-fold cross-validation (minimum criteria) was 0.039, and log (λ) was -3.245. (**B**) Regression coefficient profiles of 22 immune signatures in the LASSO model. The vertical dotted line is the optimal λ selected with 10-fold cross-validation, which resulted in 12 non-zero coefficients.

**Figure 4:**
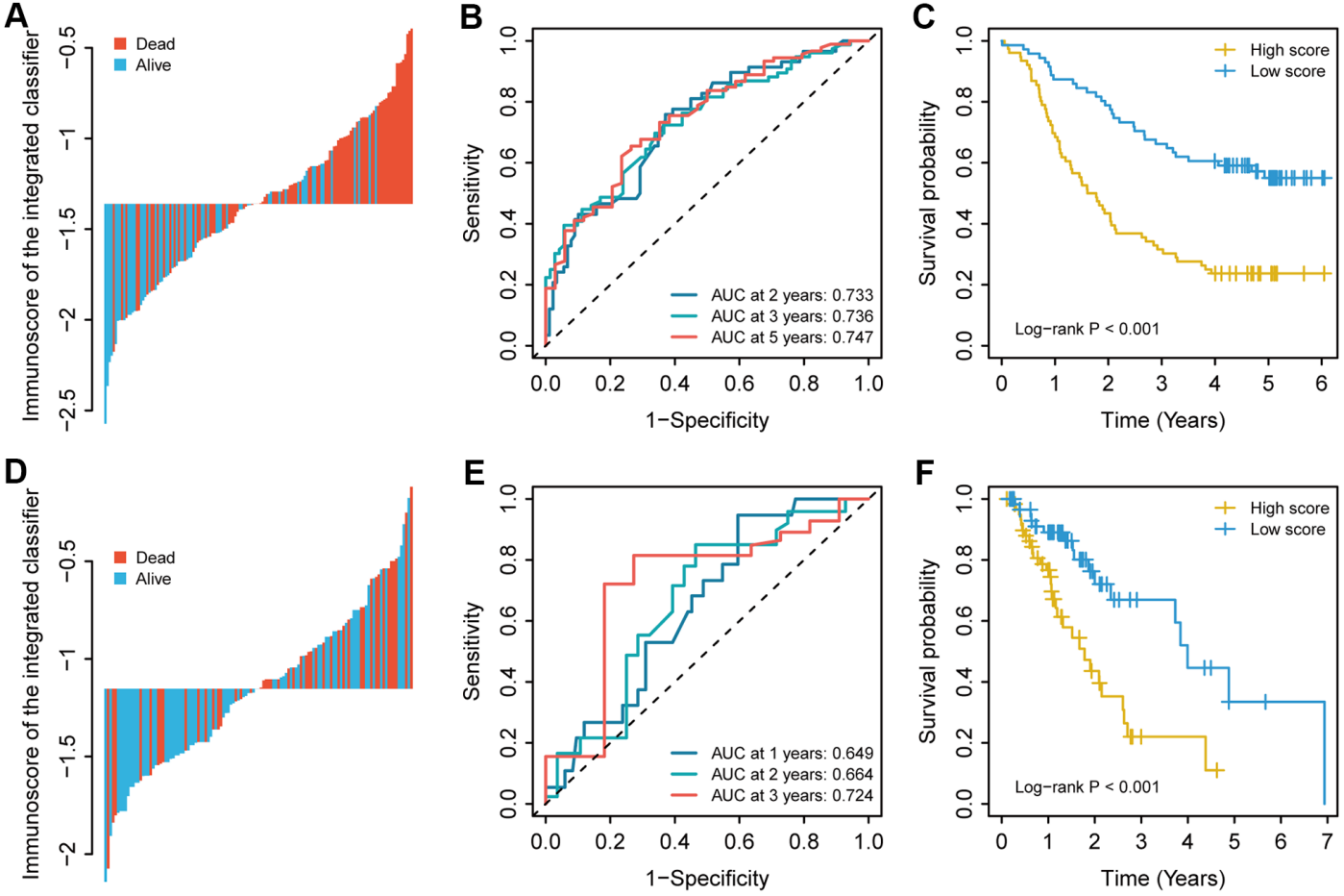
The immunoscore for each patient, the time-dependent ROC curve, and the Kaplan–Meier curves in (**A**–**C**) the training cohort and (**D**–**F**) the validation cohort. The 2-, 3-, and 5-year AUCs were used in the training cohort, and the 1-, 2-, and 3-year AUCs were used in the validation cohort to evaluate the accuracy of the prognostic model. The log-rank test was performed to analyse survival status.

**Figure 5:**
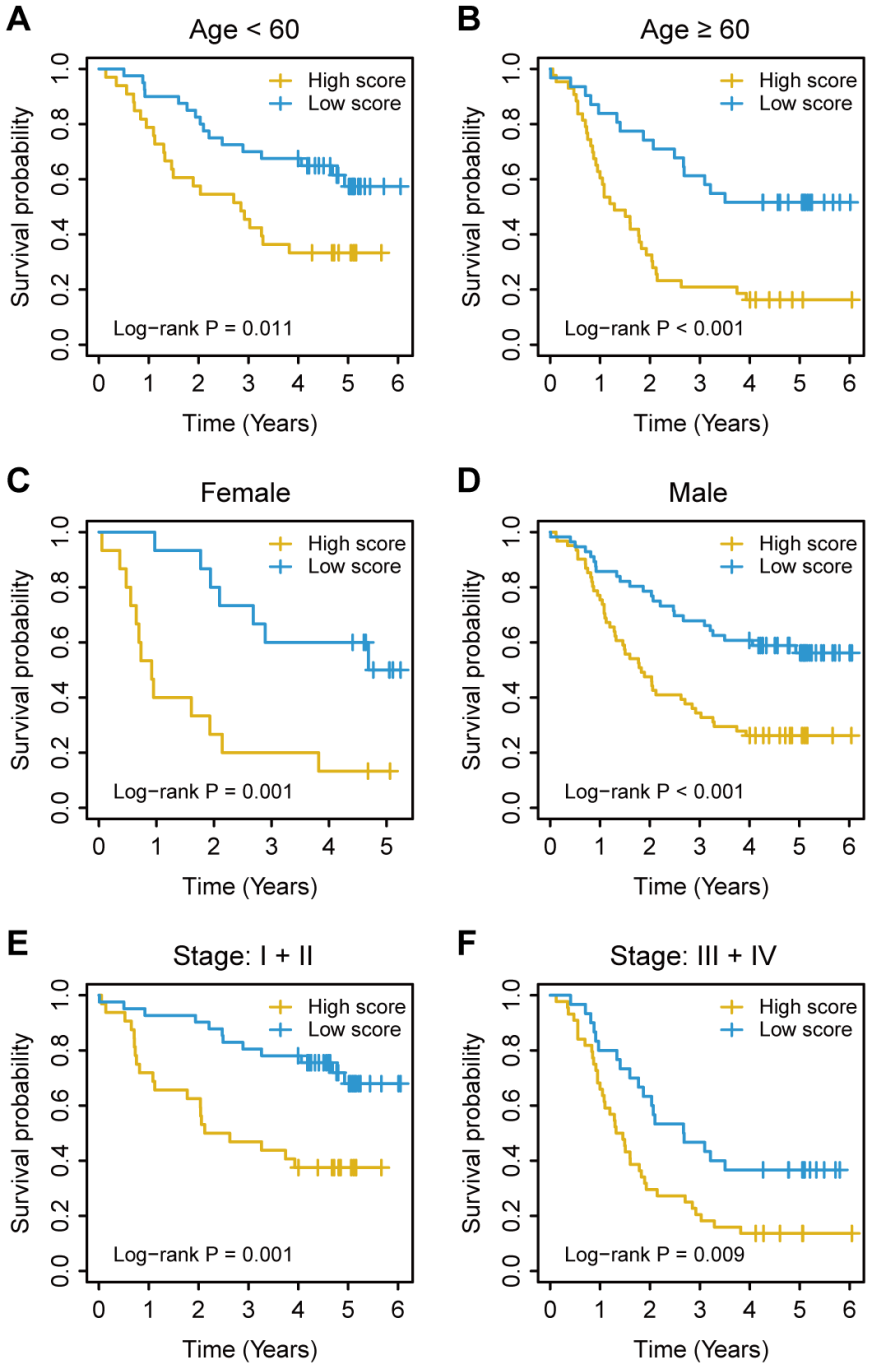
**Kaplan–Meier survival analysis based on the immunoscore signature of oesophageal cancer patients in the training cohort stratified based on clinical characteristics.** (**A**, **B**) Age. (**C**, **D**) Sex. (**E**, **F**) TNM stage. The log-rank test was performed to calculate P values.

For the training cohort, each immune cell fraction was dichotomized based on the optimal cut-off value generated by the survminer package (Supplementary Table 1). The LASSO Cox regression analysis identified 12 non-zero coefficient features for the calculation of prognostic immunoscores (Figure 3A, 3B). The immunoscore calculation formula is as follows: Immunoscore = (-0.294 × Memory B cells) + (-0.567 × Plasma cells) + (-0.257 × CD8^+^ T cells) + (0.068 × CD4^+^ Memory T cells (resting)) + (0.161 × Regulatory T cells (Tregs)) + (-0.043 × Gamma delta T cells) + (-0.410 × Monocytes) + (-0.251 × M0 Macrophages) + (-0.354 × M1 Macrophages) + (0.301 × Dendritic cells (resting)) + (-0.440 × Dendritic cells (activated)) + (-0.236 × Neutrophils), where 0 or 1 is assigned based on whether the immune cell fraction is below or above the corresponding cut-off value, respectively. For the training cohort, correlation analysis showed that a lower immunoscore was correlated with a higher survival rate of oesophageal cancer patients (Figure 4A). A time-dependent receiver operating characteristic (ROC) curve was used to evaluate the prognostic accuracy of 12 immune cell types over time. The AUC of the prognostic model was 0.733 at year 2, 0.736 at year 3, and 0.747 at year 5 (Figure 4B). Next, the training cohort was divided into a high-immunoscore group and a low-immunoscore group based on the median score (hazard ratio [HR], 2.70; 95% confidence interval [CI], 1.74-4.20; P < 0.001), which had 5-year survival rates of 23.7% (95% CI, 14.1-33.2) and 55.0% (95% CI, 43.0-67.0), respectively (Figure 4C). Stratification analysis of different clinical characteristics showed that immunoscore was significantly correlated with survival status (Figure 5 and Supplementary Table 2).

### Immunoscore validation in the TCGA cohort

To evaluate the prognostic value of the immunoscore-based prognostic model in different populations, we used the same formula to calculate the immunoscore in the validation cohort. As in the model above, a lower immunoscore was associated with a more favourable prognosis (Figure 4D). For the validation cohort, the AUCs were 0.649 at year 1, 0.664 at year 2, and 0.724 at year 3. As in the training cohort, the patients in the validation cohort were divided into a high-immunoscore group and a low-immunoscore group based on the median score (Figure 4E). The 3-year survival rates were 66.9% (95% CI, 49.7-84.1) in the low-immunoscore group and only 22.0% (95% CI, 6.1-37.9) in the high-immunoscore group (HR, 2.93; 95% CI, 1.56-5.50; P < 0.001) (Figure 4F).

Univariate and multivariate Cox regression analyses were performed with immunoscore as a continuous variable. After controlling for other clinical covariates, a significant between-group difference was still observed in the training cohort (HR, 4.78; 95% CI, 2.64-8.67; P < 0.001) and the validation cohort (HR, 2.15; 95% CI, 1.04-4.45; P = 0.040), suggesting that the immunoscore may be used as an independent predictor for the prognosis of oesophageal cancer (Table 2).

**Table 2:** Univariate and multivariate cox regression analyses of immunoscores and clinical data in different cohorts of oesophageal cancer patients.

	**Training cohort**					**Validation cohort**			
	**HR in UVA**	**P value**	**HR in MVA**	**P value**		**HR in UVA**	**P value**	**HR in MVA**	**P value**
Immunoscore	5.00 (2.91-8.59)	**< 0.001**	4.78 (2.64-8.67)	**< 0.001**		2.59 (1.35-4.98)	**0.004**	2.15 (1.04-4.45)	**0.040**
Age	1.04 (1.01-1.06)	**0.006**	1.03 (1.00-1.06)	**0.025**		0.98 (0.96-1.00)	0.107		
Sex (Female vs. male)	0.79 (0.48-1.30)	0.351				2.60 (0.92-7.31)	0.070		
Alcohol (No vs. yes)	0.88 (0.58-1.34)	0.564				0.91 (0.49-1.69)	0.760		
Tumour grade (G1)	Reference					Reference			
G2	1.06 (0.58-1.97)	0.842				1.73 (0.51-5.84)	0.379		
G3	1.56 (0.81-3.01)	0.188				1.49 (0.42-5.24)	0.533		
T stage	1.43 (0.84-2.43)	0.184				0.95 (0.50-1.82)	0.886		
N stage	1.88 (1.15-3.08)	0.012	1.54 (0.86-2.75)	0.146		2.59 (1.07-6.30)	0.035	1.25 (0.48-3.24)	0.645
tumour stage (I + II vs. III + IV)	2.56 (1.65-3.96)	**< 0.001**	1.75 (1.05-2.90)	**0.031**		3.64 (1.82-7.28)	**< 0.001**	3.26 (1.54-6.89)	**0.002**

HR, hazard ratio; UVA, univariate analysis; MVA, multivariate analysis.

### Nomogram plotting and validation

**Figure 6:**
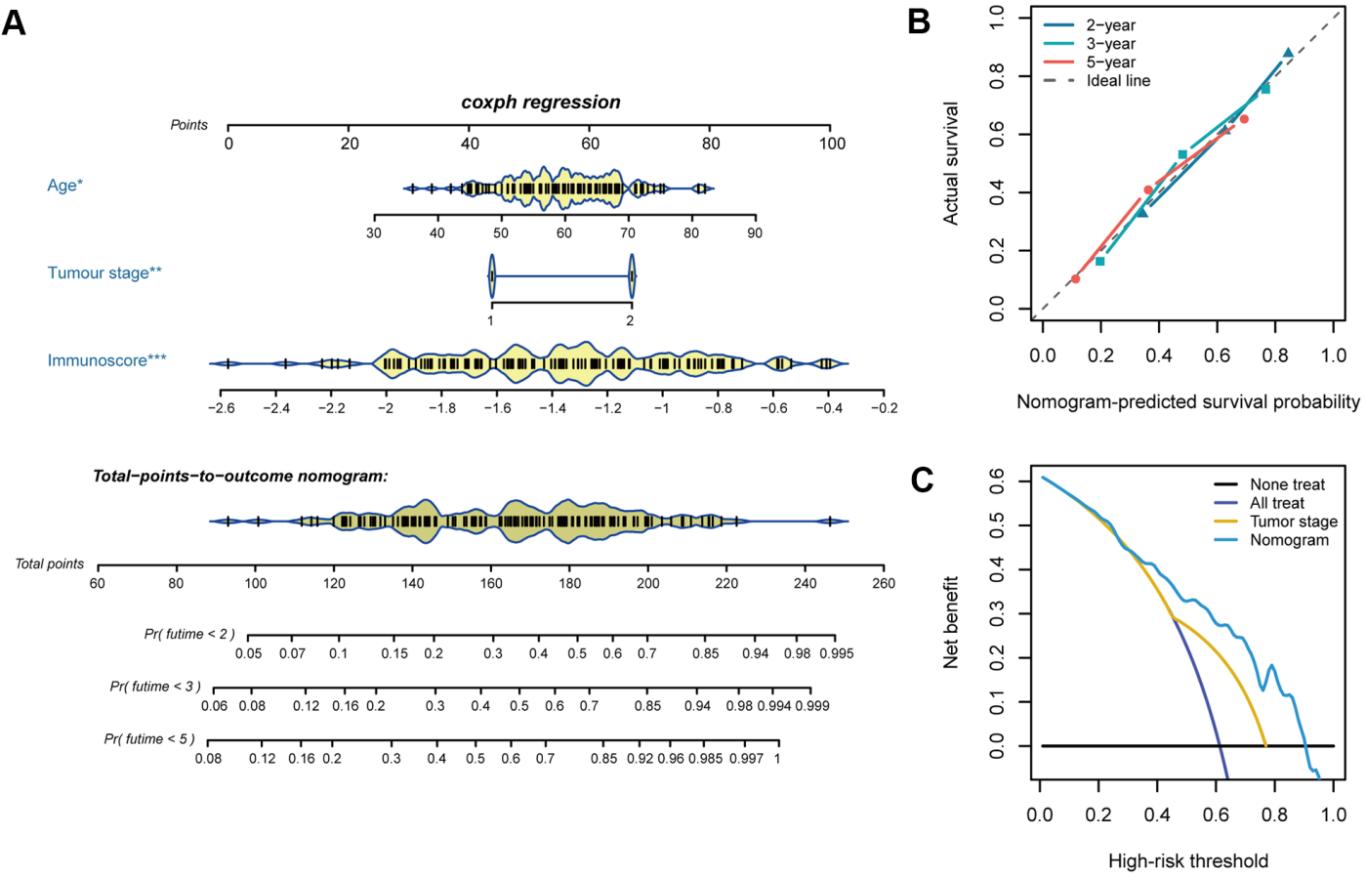
**Nomogram plotting and evaluation.** (**A**) Clinical characteristics and immunoscores were used to develop a nomogram for prediction of the 2-, 3-, and 5-year survival rates of oesophageal cancer patients. (**B**) The calibration curve demonstrated agreement between the predictive and observed outcomes for 2-, 3-, and 5-year survival. The 45-degree dashed line indicates a perfect prediction of the ideal model, while the dotted lines indicate the actual performance of the nomogram. The closer the dotted line matches the dashed line, the better the prediction accuracy. (**C**) Decision curve analysis of the nomogram and tumour stage for the 5-year risk among patients with oesophageal cancer.

Cox regression analysis was performed to determine the immunoscore, which showed that age and tumour stage were independent predictors of the prognosis of oesophageal cancer. For quantitative prognostic predictions, we used the training cohort to build a model containing the above independent predictors, which is presented as a nomogram (Figure 6A). The calibration curve demonstrated good agreement between the prediction model and the ideal model for the prognosis of oesophageal cancer (Figure 6B). The C-index of the nomogram was 0.718 (95%, 0.668-0.768) in the training cohort. The C-index of the prediction model was 0.716 (95%, 0.612-0.820) in the validation cohort. Decision curve analysis showed that when the threshold probability was greater than 0.3, the use of the plotted nomogram for prognostic prediction had more net benefits than indiscriminate treatment or no treatment, suggesting that the nomogram was clinically useful (Figure 6C).

### Biological functions underlying the immunoscore

**Figure 7:**
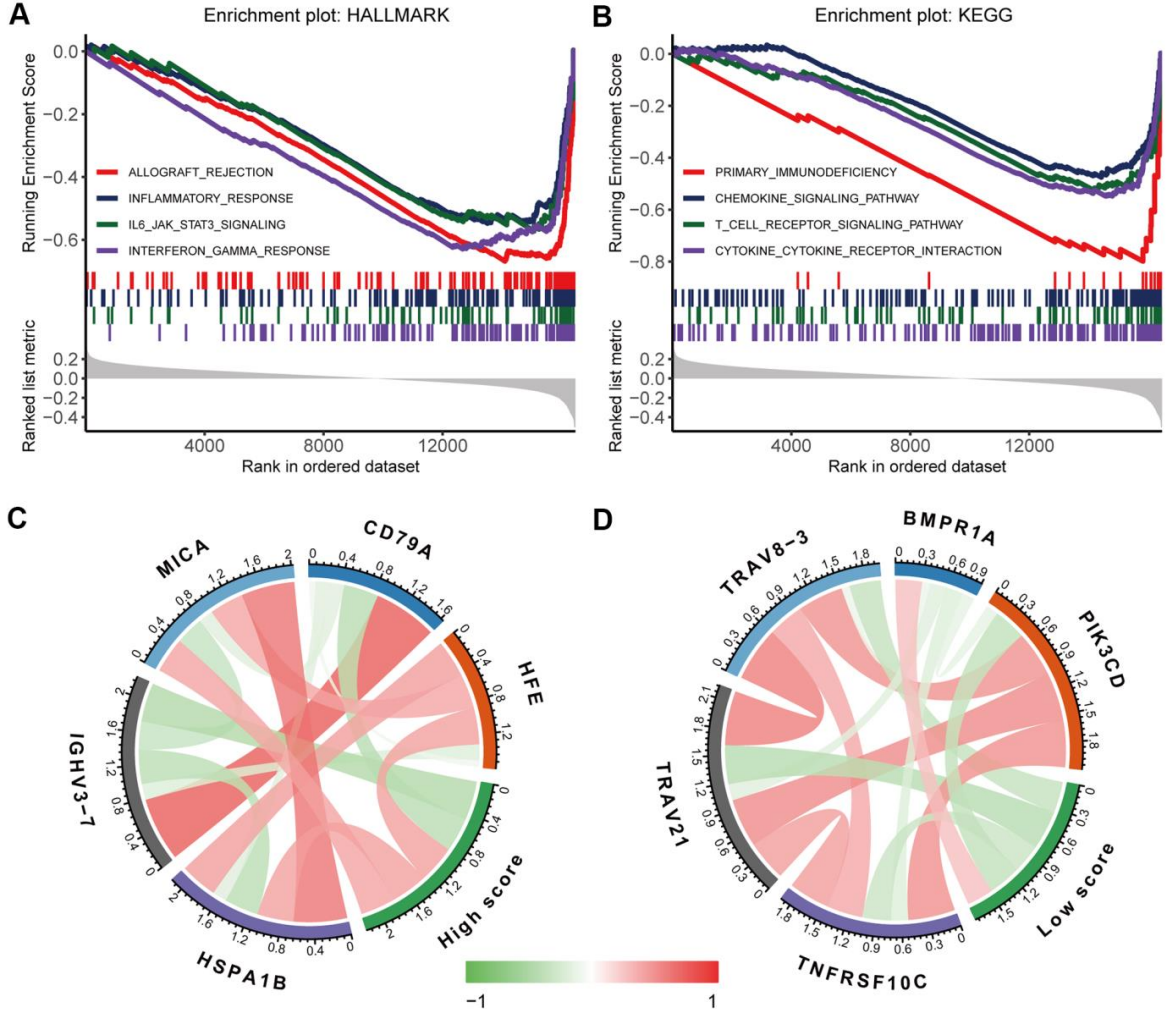
Biological functions underlying the immunoscore. GSEA of (**A**) h.all.v7.1.symbols and (**B**) c2.cp.kegg.v7.1.symbols identified the most significant pathways and processes related to a low immunoscore. Correlation analysis of (**C**) a high immunoscore and (**D**) a low immunoscore with immune-related gene expression. The red line indicates a positive correlation, and the green line indicates a negative correlation. A darker colour corresponds to a stronger correlation.

To further investigate the biological functions underlying the immunoscore, we divided the oesophageal cancer patients into the high-immunoscore group and the low-immunoscore group and then performed gene set enrichment analysis (GSEA) to identify potential biological pathways based on the gene expression data of oesophageal cancer. Many immune-related pathways, such as the inflammatory response, IL-6-JAK-STAT3 signalling pathway, interferon-gamma response, chemokine-signalling pathway, T-cell receptor signalling pathway, and cytokine–cytokine receptor interaction pathways, were significantly enriched in the low-immunoscore group (false discovery rate (FDR) < 0.05 for all; Figure 7A, 7B). Correlation analysis of the immunoscore signature and immune-related gene expression showed that a high immunoscore was significantly correlated with the expression of antigen processing and presentation and B-cell receptor signalling pathway genes, while a low immunoscore was correlated with the expression of T-cell receptor signalling pathway genes and cytokine receptors (Figure 7C, 7D and Supplementary Table 3). These high correlations with immune-related pathways provide clues for further molecular mechanism studies related to the immunoscore.

## DISCUSSION

In this study, we built a novel prediction model based on a signature of 12 immune cell types to improve survival predications after a diagnosis of oesophageal cancer. The results showed a significant difference in the overall survival between the high-immunoscore group and the low-immunoscore group. After controlling for confounding clinical characteristics, multivariate analysis showed that immunoscore was still an independent prognostic factor, which was further validated in an independent cohort. These data indicate that immunoscore has similar predictive efficacy as conventional predictors.

Previous studies on the potential mechanisms of oesophageal cancer have focused on tumour cell-intrinsic features [13–15]. Tumour cells are in a complex microenvironment, which is critical for tumour cell survival and plays an important role in the proliferation, invasion, and metastasis of oesophageal cancer [16, 17]. The tumour microenvironment contains many non-tumour cells, including different subtypes of tumour-infiltrating immune cells, and the interaction of these cells determines the balance of their oncogenic/anticancer effect [18]. Some researchers have performed immunohistochemistry to quantify infiltrating immune cells and evaluate their role in oesophageal cancer progression and treatment responses [5, 6, 19, 20], and others have used the immunoprofile to build models to predict postoperative survival [21]. These studies mainly used immunohistochemistry to analyse immune cells, but immunohistochemistry relies on the identification of cell-surface markers and cannot comprehensively assess different immune cell types or effectively distinguish closely related cell populations. In addition, the selection of visual fields on a slide is highly subjective, with low intra-rater and inter-rater reproducibility. These factors have limited the sample size and the number of cell types in previous studies.

Unlike previous studies, this study investigated the infiltration pattern of immune cells in oesophageal cancer by using CIBERSORT to estimate infiltrating immune cell fractions in oesophageal cancer tissue and normal tissue based on the gene expression data of oesophageal cancer from public databases. CIBERSORT uses the expression profiles of 22 purified leukocyte subpopulations to define gene expression in immune cells and effectively distinguish these cell types without the need for cell type-specific markers. It characterizes the relative immune cell fraction by using the complex gene expression profile in the tissue. In this study, we found significant differences in the abundance of different immune cells between tumour tissue and normal tissue. In addition, the LASSO Cox regression model was used to evaluate the correlation between immune cell profile and prognosis and select 12 potential predictors from 22 candidate cell sets. Unlike other methods that select predictors based on univariate correlation, the LASSO Cox regression model generates the immunoscore signature based selected immune cell sets and thus significantly improves predictive accuracy. Recent studies have used this strategy to integrate multiple features into a single variable. For example, Li et al [22] built a new prognostic model for oesophageal cancer based on eight lncRNAs, and Huang et al [23] used the radiomics signature as a preoperative predictor of lymph node metastasis in patients with colorectal cancer. Similarly, this study demonstrated good predictive efficacy of the immunoscore signature that combined multiple immune features, in both the training cohort and the validation cohort. Stratified analysis showed that immunoscore may be used to divide patients with the same TNM stage or other clinical characteristics into the high-risk group and the low-risk group, with significantly different survival probabilities. This finding may help clinicians make decisions about adjuvant therapy in high-risk patients to improve patient outcomes.

In addition, we built a nomogram based on immunoscore and significant clinical characteristics, which may be used for individualized prediction of survival probability. The most important use of nomograms is to decide on additional clinical treatment or care for individual patients. Methods based on accuracy evaluation, such as ROC analysis, discrimination, and calibration, cannot evaluate the clinical consequences of their predictions. To investigate clinical usefulness, we performed decision curve analysis to evaluate the efficacy of nomogram-based decision-making in improving patient outcomes. The results showed that when the threshold probability was greater than 0.3, the decision based on the immunoscore nomogram was associated with more clinical benefit to patients.

This study has some limitations. First, the data sets used for the prognostic model were retrieved from public databases that have limited clinical information. Therefore, some patients with acute infections or immune disorders may have been included in the study and skewed the results. Second, many factors are related to the progression of oesophageal cancer. We were unable to use potential risk factors (such as genetic history, occupation, dietary habits, and environmental exposure) to improve predictive accuracy due to incomplete information. Third, this was a retrospective study with a small sample size, so researchers should exercise caution in interpreting our data.

In summary, we used CIBERSORT to estimate the infiltration pattern of immune cells in oesophageal cancer based on gene expression data and built a nomogram that included immunoscore, age, and tumour stage for the prediction of the overall survival of oesophageal cancer patients. This method provides new directions for investigating the relationship between immune cells and tumours, tumour treatment, and the search for efficacy measures.

## MATERIALS AND METHODS

### Data source

The expression profile and clinical data of oesophageal cancer were retrieved and downloaded from the public (https://www.ncbi.nlm.nih.gov/geo/) database with a query of GSE53625 [24]. The expression data were generated with the Agilent human lncRNA + mRNA array v2.0 platform. Before the analysis, we re-annotated Agilent array probes. The probe sequences were aligned with SeqMap [25] from the GENCODE database (GRCh38, release 29) [26]. Only probes that were uniquely mapped to the genome with no mismatch were retained. Validation was performed on an independent cohort of oesophageal cancer patients from TCGA. The high-throughput sequencing (HTSeq)-fragments per kilobase of transcript per million mapped reads (FPKM) transcriptome data of the TCGA-ESCA project and survival information were obtained from Genomic Data Commons through the R package TCGAbiolinks [27].

### Estimation of immune cell abundance

We used CIBERSORT to estimate the fractions of different immune cells in tissues based on the reference signature (LM22) matrix of 547 genes [10]. We uploaded the gene expression data prepared based on the example mixture file to the CIBERSORT web portal (https://cibersort.stanford.edu/) and ran the program with the default LM22 feature matrix at 1000 permutations. Only samples with significant CIBERSORT global deconvolution (P < 0.05) were included in subsequent analyses. For a given mixture sample, the relative fraction of immune cells was calculated with CIBERSORT and may be used directly for comparisons between immune cells or between studies.

The main results from CIBERSORT were validated with xCell (https://xcell.ucsf.edu/), a new proven method for counting cell subpopulations from a tissue expression profile [28]. xCell is a gene signature-based method and performs single-sample gene set enrichment analysis (ssGSEA) from the gene expression data for 64 immune and stromal cells.

### Gene set enrichment analysis

To investigate biological pathways related to the immunoscore signature, we performed GSEA using the R clusterProfiler package on the gene expression profile [29]. The h.all.v7.1.symbols.gmt and c2.cp.kegg.v7.1.symbols reference gene set files used in this study were downloaded from the Molecular Signature Database (https://www.gsea-msigdb.org/gsea/index.jsp). Gene sets with an adjusted P < 0.05 after 1000 permutations were considered significantly enriched.

### Statistical analysis

SPSS 22.0 and R 3.6.2 were used for statistical analysis. All statistical tests were two-tailed, and P < 0.05 was considered statistically significant. Count data were analysed with the chi-squared test or Fisher’s exact test. Normally distributed measurement data were analysed with the independent t-test, and non-normally distributed measurement data were analysed with the Mann–Whitney U test. To control for the FDR, the Benjamini-Hochberg method was used to adjust the P value when comparing immune cell fractions between the cancer group and the control group. Pearson correlation analysis was performed to identify any correlations between subsets of immune cells, and the corrplot package was used to visualize the resulting correlation matrix. The least absolute shrinkage and selection operator (LASSO) Cox regression method was used to screen the most significant immune cells for the prognosis of oesophageal cancer [30]. In the LASSO model, all immune cell fractions were dichotomized based on the optimal cut-off value calculated by the survminer package. The Kaplan–Meier method was used to analyse survival rates, and the log-rank test was used to analyse survival time. The time-dependent ROC curve was used to analyse the sensitivity and specificity of immunoscore-based survival predictions, and the area under the ROC curve (AUC) was quantified with the timeROC package [31]. The Cox proportional hazards model was used for univariate and multivariate prognostic analyses, and the regplot package was used to plot a nomogram for statistically independent predictors. The performance of the nomogram was evaluated with the calibration curve and Harrell's concordance index (C-index). In addition, decision curve analysis was performed by quantifying the net benefits of different threshold probabilities in evaluating clinical usefulness [32, 33].

## Supplementary Material

Supplementary Tables
