# Supplementary material for: An immunoscore nomogram for predicting survival in patients with oesophageal cancer
Source: Aging (Albany NY). 2021 Mar 10;13(6):8762–76. doi: 10.18632/aging.202686 (PMC8034926; doi:10.18632/aging.202686)
Supplement: Supplementary Tables [file aging-13-202686-s001.pdf]

## SUPPLEMENTARY TABLES

**Supplementary Table 1. Cut-off values of different immune cells.**

| Immune cell type | Cut-off value |
|---|---|
| Naive B cells | 0.047 |
| Memory B cells | $8.0*10^{-4}$ |
| Plasma cells | 0.147 |
| $CD8^+$ T cells | 0.121 |
| $CD4^+$ naive T cells | 0 |
| $CD4^+$ memory T cells (resting) | 0.003 |
| $CD4^+$ memory T cells (activated) | 0.013 |
| Follicular helper T cells | 0.071 |
| Regulatory T cells (Tregs) | 0.071 |
| Gamma delta T cells | 0 |
| NK cells (resting) | 0 |
| NK cells (activated) | 0.008 |
| Monocytes | 0.024 |
| M0 Macrophages | 0.013 |
| M1 Macrophages | 0.039 |
| M2 Macrophages | 0.073 |
| Dendritic cells (resting) | 0.178 |
| Dendritic cells (activated) | 0.009 |
| Mast cells (resting) | 0.03 |
| Mast cells (activated) | 0.127 |
| Eosinophils | 0 |
| Neutrophils | $8.0*10^{-4}$ |

**Supplementary Table 2. Univariate cox regression analysis of immunoscore in the training cohort stratified based on clinical characteristics.**

| Subgroup | HR (95% CI) | P value |
|---|---|---|
| Age (years) | | |
| <60 | 4.37 (1.95-9.80) | < 0.001 |
| ≥60 | 5.14 (2.40-10.98) | < 0.001 |
| Sex | | |
| Female | 6.76 (2.36-19.39) | < 0.001 |
| Male | 4.74 (2.52-8.93) | < 0.001 |
| Pathologic tumour stage | | |
| I + II | 9.86 (3.35-29.06) | < 0.001 |
| III + IV | 2.80 (1.47-5.32) | 0.002 |

Note: Immunoscore was used as a continuous variable in regression analysis.

**Supplementary Table 3. Correlation analysis of immunoscore and gene expression in the high-immunoscore group and low-immunoscore group of the training cohort.**

| | Gene | High-immunoscore group | | | Low-immunoscore group | | |
|---|---|---|---|---|---|---|---|
| | | r | 95% CI | P value | r | 95% CI | P value |
| Antigen Processing and Presentation | *HFE* | 0.45 | 0.25~0.61 | < 0.001 | -0.24 | -0.45~-0.01 | 0.041 |
| Antigen Processing and Presentation | *HSPA1B* | 0.44 | 0.24~0.61 | < 0.001 | 0.00 | -0.24~0.23 | 0.979 |
| Antigen Processing and Presentation | *HLA-E* | 0.39 | 0.18~0.57 | < 0.001 | -0.02 | -0.25~0.21 | 0.859 |
| Antigen Processing and Presentation | *PSMC6* | 0.42 | 0.22~0.59 | < 0.001 | 0.03 | -0.20~0.26 | 0.785 |

| | | | | | | | |
|---|---|---|---|---|---|---|---|
| Antigen Processing and Presentation | *MICA* | 0.43 | 0.22~0.60 | < 0.001 | 0.05 | -0.18~0.28 | 0.660 |
| Antigen Processing and Presentation | *HSP90AB1* | 0.41 | 0.21~0.58 | < 0.001 | 0.06 | -0.18~0.29 | 0.640 |
| B-cell receptor Signaling Pathway | *IGHV3-7* | -0.47 | -0.63~-0.28 | < 0.001 | -0.25 | -0.45~-0.01 | 0.038 |
| B-cell receptor Signaling Pathway | *CD79A* | -0.42 | -0.59~-0.22 | < 0.001 | -0.21 | -0.43~0.02 | 0.072 |
| B-cell receptor Signaling Pathway | *IGLV5-37* | -0.42 | -0.59~-0.21 | < 0.001 | 0.00 | -0.24~0.23 | 0.976 |
| B-cell receptor Signaling Pathway | *IGLV1-36* | -0.41 | -0.58~-0.20 | < 0.001 | -0.18 | -0.40~0.05 | 0.129 |
| B-cell receptor Signaling Pathway | *PIK3CD* | 0.18 | -0.04~0.39 | 0.114 | -0.36 | -0.55~-0.14 | 0.002 |
| Cytokine Receptors | *TACR1* | -0.40 | -0.58~-0.20 | < 0.001 | 0.02 | -0.22~0.25 | 0.885 |
| Cytokine Receptors | *TNFRSF10C* | 0.15 | -0.08~0.36 | 0.210 | -0.32 | -0.52~-0.10 | 0.006 |
| Cytokine Receptors | *NR4A3* | 0.11 | -0.11~0.33 | 0.327 | -0.31 | -0.51~-0.09 | 0.008 |
| Cytokine Receptors | *BMPR1A* | -0.15 | -0.36~0.08 | 0.194 | 0.30 | 0.07~0.50 | 0.011 |
| Cytokine Receptors | *TNFRSF9* | -0.03 | -0.26~0.19 | 0.773 | -0.33 | -0.52~-0.10 | 0.005 |
| T-cell receptor signaling Pathway | *TRAV8-3* | 0.00 | -0.23~0.22 | 0.974 | -0.35 | -0.54~-0.13 | 0.003 |
| T-cell receptor signaling Pathway | *TRAV21* | -0.13 | -0.35~0.10 | 0.259 | -0.44 | -0.61~-0.23 | < 0.001 |
| T-cell receptor signaling Pathway | *TRBV19* | -0.11 | -0.33~0.12 | 0.340 | -0.39 | -0.57~-0.18 | < 0.001 |
| T-cell receptor signaling Pathway | *TRAV1-2* | -0.19 | -0.40~0.03 | 0.094 | -0.46 | -0.62~-0.25 | < 0.001 |
